# Bayesian supervised learning and state estimation in a model of the cerebellum

**DOI:** 10.1186/1471-2202-16-S1-P12

**Published:** 2015-12-18

**Authors:** Benjamin Campbell

**Affiliations:** 1 Laboratory of Biological Modeling, The Rockefeller University, New York, NY, 10065, USA

## 

The last decade has seen an explosion of research on the topic of Bayesian inference in neural systems. The cerebral cortex is now widely believed to form predictive internals models of the environment. But what does this mean for the cerebellum, which has historically been attributed just this function? And what might Bayesian inference mean in the cerebellar context?

I present a cerebellar model unifying the Bayesian framework with classical cerebellar theories of supervised learning, state estimation, and control theory. This model builds on the dominant supervised learning theory of cerebellar function, which traces to the pioneering work of Marr. This model is extended to account for Bayesian optimal updating of the uncertainties inherent in probabilistic inference. Further, the model is capable of learning about states with little to no supervised input. It also provides theoretical explanation for a wide range of phenomenology observed of the circuit -- most importantly unsupervised synaptic plasticity upstream of cerebellar Purkinje cells.

The connectionist architecture of this model is closely based on that of the cerebellum. In the input stage, unsupervised learning in the cerebellar granular layer is seen as essential for subsequent supervised learning in the Purkinje layer. Based on Golgi cell feedback, the granular layer learns to encode supervised learning covariances in a manner akin to the kernel learning of Gaussian processes. This circuit then predicts and filters mossy fiber input, conveying only select signals downstream for supervised learning. This granular representation is further influenced by a global serotonergic signal conveyed by Lugaro cells. In the cerebellar molecular layer, inhibitory interneurons then provide higher-order corrections to these estimates. Finally, learning in the cerebellar nuclei is seen as a parallel, simpler, regression model, allowing for faster, courser adjustments, corrected in time by the cerebellar cortical circuit.

